# Effects of Exercise Training on Muscle Function

**DOI:** 10.3390/life15101632

**Published:** 2025-10-20

**Authors:** Marco Duca, Athos Trecroci

**Affiliations:** 1 Department of Exercise and Sport Science, East Tennessee State University, Johnson City, TN 37614, USA; 2 Department of Biomedical Sciences for Health, Università degli Studi di Milano, 20122 Milano, Italy; athos.trecroci@unimi.it

Exercise training is a versatile and powerful tool to improve muscle function. Its diverse applications allow for enhancing physical qualities across various populations, from chronically ill patients to youth engaged in recreational sports. In fact, the role of exercise in both preventing disease [1,2] and improving performance [3,4] is well established. More recently, exercise has been recognized as a powerful adjunct to conventional treatments for mitigating symptoms of various illnesses [5,6]. This Special Issue addresses these themes through seven original research articles and two reviews, highlighting two primary domains of exercise implementation: clinical setting and athletic performance.

In the clinical context, five original studies examine the effects of exercise on reducing postural disorders in children, lowering cardiovascular risk in adults, improving symptoms in fibromyalgia and stroke, and the selection ofoptimizing cuff pressure for blood flow restriction training as a therapeutic modality. On the athletic performance side, two systematic reviews examine high-intensity interval training (HIIT) as a stimulus relevant to cardiovascular–metabolic demands and neuromuscular expression, alongside resistance training as the primary driver of force and hypertrophy. One study focuses on the intermittent versus continuous small-sided games (SSGs) as field-based levers to organize on-pitch intensity and workload, while another evaluates Pilates and yoga as distinct approaches targeting balance and movement quality.

Exercise also plays a key role in prevention and complements therapeutic interventions. Endurance training in untrained men improves antioxidant defenses and hormonal balance [7], adaptations that likely contribute to reducing cardiovascular risk. Postural disorders in children are linked to sedentarism and poor ergonomics [8], emphasizing the importance of early interventions that integrate physical activity into daily routines.

Clinical populations can benefit equally from exercise interventions. For example, in patients with fibromyalgia, a combination of aerobic training and Pilates proves superior to physical modalities for managing pain [9], highlighting the value of movement-based therapies. In stroke rehabilitation, botulinum toxin combined with exercise outperforms physical modalities when paired with a stretching program [10], demonstrating that pharmacological and exercise-based modalities can be implemented synergistically to promote recovery. An exercise modality that is now commonplace in athletic performance and rehabilitation alike is blood flow restriction training. Practitioners can now take advantage of newly developed regression equations to select the appropriate limb occlusion pressure based on anthropometric measurements [11].

Regarding athletic performance, one review explores the neuromuscular benefits of HIIT, highlighting how it enhances strength and power through improved motor-unit recruitment, a shift toward fast-twitch and hybrid type muscle fibers, and metabolic changes that support high-effort repetition. However, HIIT should not be used alone when aiming to maximize strength or muscle size. According to Hung et al. [12], HIIT is most effective when combined with structured resistance and plyometric training within a well-planned, periodized program (i.e., performed separately from or after strength training) and recovery strategies (i.e., active recovery). Another review focuses on female team-sport athletes. Čaprić et al. [13] summarize the effects of various HIIT protocols on physical performance in female basketball players, reporting consistent improvements in aerobic fitness, change of direction speed, sprint capacity, lower-body power, and body composition. Despite the wide range of training approaches in the literature, the consensus supports smart, seasonal integration of HIIT with intensity and workload tailored to the competitive calendar.

This Special Issue also emphasizes the comparison between intermittent and continuous sport-specific activities in young male team-sport athletes. Over four weeks, both formats improve agility, horizontal jumping, aerobic and anaerobic power, and balance in young soccer players, though the continuous group reports a higher perceived rate of exertion than the intermittent group [14]. Coaches can use these findings to select SSG formats based on session objectives, weekly training load, and player readiness rather than physical performance alone.

In a different study, an eight-week intervention compares the effects of Pilates and yoga on dynamic balance and movement quality in adult female fencers. Both exercise modalities, practiced twice weekly, elicit a remarkable increase in the balance composite scores (measured via the lower-quarter Y-Balance Test) and functional movement scores (FMS^®^), with no significant differences between them [15]. Accordingly, the choice between Pilates and yoga may depend on the athlete’s circumstances and preferences, given their similar benefits for dynamic balance and movement quality.

Exercise emerges as a powerful modality to prevent and mitigate pathologies. Specific training interventions can be safely implemented in diverse clinical populations to both prevent the insurgence of diseases and improve symptoms alongside traditional treatments. In this context, exercise can truly be considered a form of medicine. Moreover, coaches and practitioners can benefit from the current evidence on planning general and sport-specific training by appropriately dosing HIIT, structuring match-like sessions with intermittent and continuous SSGs, and incorporating supportive modalities (i.e., Pilates/yoga). This approach aligns with evidence-based practice, where general and sport-specific elements coexist within the same training program [16].
